# Homoharringtonine Inhibits CVS-11 and Clinical Isolates of Rabies Virus In Vitro: Identified via High-Throughput Screening of an FDA-Approved Drug Library

**DOI:** 10.3390/v17070945

**Published:** 2025-07-04

**Authors:** Kalenahalli Rajappa Harisha, Varun Kailaje, Ravinder Reddy Kondreddi, Chandra Sekhar Gudla, Shraddha Singh, Sharada Ramakrishnaiah, Shrikrishna Isloor, Shridhar Narayanan, Radha Krishan Shandil, Gudepalya Renukaiah Rudramurthy

**Affiliations:** 1 Foundation for Neglected Disease Research (FNDR), Bengaluru 561203, India; harisha.r@fndr.in (K.R.H.); varun.k@fndr.in (V.K.); ravinder.kondreddi@fndr.in (R.R.K.); chandrasekhar.g@fndr.in (C.S.G.); shridhar.narayanan@fndr.in (S.N.); rk.shandil@fndr.in (R.K.S.); 2 Veterinary College, Karnataka Veterinary, Animal and Fisheries Sciences University (KVAFSU), Hebbal, Bengaluru 560024, India; shraddhabrh@gmail.com (S.S.); sharadadr@yahoo.co.in (S.R.); kisloor@gmail.com (S.I.)

**Keywords:** clinical isolate, CVS-11, flow cytometry, high-throughput assay, homoharringtonine, RABV

## Abstract

Rabies, a viral encephalitis caused by rabies virus (RABV), is 100% fatal upon the onset of symptoms. Effective post-exposure prophylaxis (PEP) measures are available, but they are often difficult to access in low-income countries. WHO estimates about 59,000 deaths due to rabies globally, and the majority are contributed by developing countries. Hence, developing drugs for the treatment of post-symptomatic rabies is an urgent and unmet demand. It is worth noting that previous efforts regarding antiviral strategies, such as small-interfering RNA, antibodies and small-molecule inhibitors, against the rabies virus have failed to show efficacy in pre-clinical studies, especially when the virus has reached the central nervous system (CNS). Therefore, drug repurposing seems to be an alternative tool for the development of new anti-rabies drugs. We validated and used a high-throughput, FITC-conjugated antibody-based flow cytometry assay to expedite the identification of repurposable new drug candidates against the RABV. The assay was validated using ribavirin and salinomycin as reference compounds, which showed EC50 values of 10.08 µM and 0.07 µM, respectively. We screened a SelleckChem library comprising 3035 FDA-approved compounds against RABV (CVS-11) at 10 µM concentration. Five compounds (clofazimine, tiamulin, difloxacin, harringtonine and homoharringtonine) were active against RABV, with greater than 90% inhibition. Homoharringtonine (HHT) identified in the present study is active against laboratory-adapted RABV (CVS-11) and clinical isolates of RABV, with an average EC50 of 0.3 µM in both BHK-21 and Neuro-2a cell lines and exhibits post-entry inhibition.

## 1. Importance

The FITC-conjugated antibody based high-throughput flow cytometry assay developed in the present study is highly robust for screening large chemical libraries against RABV. The assay is economical and has a shorter turnaround time than the conventional direct fluorescent antibody (DFA) assay. The repurposed drug, HHT, identified in the present screening, showed antiviral activity against laboratory adapted and clinical isolates of RABV in different cell lines, including neural cell lines such as Neuro-2a. A pre-clinical evaluation of HHT in animal infection models will help develop a repurposed drug against rabies. In addition, prior clinical use and a reasonable safety profile in humans with potent in vitro activity of HHT against RABV in various cell lines suggest HHT to be a good candidate for repurposing.

## 2. Introduction

Rabies is caused by a non-segmented, single-stranded, negative-sense RNA virus, generally termed rabies virus (RABV), leading to progressive zoonotic encephalitis. RABV belongs to the family *Rhabdoviridae* and genus *Lyssavirus* (*Mononegavirales* order). The fatality rate due to rabies is the highest among any etiological agent after the onset of symptoms. The highest case fatality due to rabies is observed mainly in Africa and Asia, where canine rabies remains endemic. Mortality due to rabies accounts for nearly 59,000 deaths per year globally, and most rabies (95%) cases occur in Asia and Africa, with 40% of these cases constituting children under the age of 15 years [1,2]. Currently, the primary source of rabies is dogs, which accounts for ~99% of fatal rabies in humans [2,3]

Rabies is a vaccine-preventable disease. Immediate and effective PEP measures of rabies immune globulins (RIGs) injection and vaccination can prevent rabies development. However, in developing countries, PEP is highly limited due to several factors such as lack of education and awareness, negligence, poverty, limited access, and the high cost of improved vaccines. Hence, rabies remains a grave public health concern in developing and underdeveloped countries. Furthermore, the currently available vaccines do not protect against phylogroup II lyssavirus infections, which comprise genotypes 2 (Lagos bat virus) and genotypes 3 (Mokola virus) [4,5].

Upon the onset of clinical symptoms, rabies is 100% fatal [6]. There are no effective therapeutic strategies to treat post-symptomatic rabies patients. A treatment strategy consisting of supportive therapy called the Milwaukee protocol was used successfully to treat clinical rabies in a 15-year-old girl in the USA [7]; however, the therapy has become questionable after a series of failures [8,9,10]. There are at least 64 reports of failures with the Milwaukee protocol [11], and the rare rabies survivors, who received a rabies vaccine (before the onset of symptoms) and treatment with the Milwaukee protocol, showed severe neurological sequelae [12]. However, there are reports of 34 rabies survivors without the Milwaukee protocol, but who received critical care management, including many from India [11,12]. In addition, ribavirin and amantadine, the main antiviral components of the Milwaukee protocol, showed no efficacy in mouse models despite their in vitro antiviral activity [13,14]. Continued use and dependency on the failed Milwaukee protocol over two decades is indirectly contributing to delaying the development of effective antiviral therapy for human rabies and moving beyond the failed Milwaukee protocol [12]. Hence, there is an urgent and unmet medical need for the development of new antiviral therapies for rabies. Research efforts have been underway over the years to develop antivirals against RABV. Several different antiviral strategies, including small-interference RNA (siRNA) [15,16,17], a monoclonal antibody [18], and small-molecule inhibitors [19,20], have been investigated and are in the early stages of drug discovery. Although considerable efforts have been made over many years, no effective antiviral has been approved for rabies treatment. This underscores the need for a new antiviral screening approach and an urgent medical need to develop potent antiviral molecules against rabies to treat post-symptomatic rabies.

The development of easily accessible antiviral screening platforms and high-throughput assays will significantly accelerate the development of antivirals against rabies. As with many other infectious diseases, drug repurposing is a key strategy to expedite the development of antivirals against rabies. In earlier studies, repurposed drugs have been screened against RABV for in vitro activity using laboratory-adapted and reporter viruses using focus forming and high-throughput assay [21,22]. In the present study, a FITC-conjugated anti-rabies antibody based flow cytometry assay is developed and validated for high-throughput antiviral screening against RABV. The assay was validated using ribavirin and salinomycin as reference compounds. It was subsequently employed to screen a repurposed, FDA-approved compound library (SelleckChem) consisting of diverse molecules that had been approved or were in different phases (I/II) of clinical trials.

The SelleckChem compound library was screened against the laboratory-adapted RABV strain, challenge virus standard-11 strain (CVS-11), in BHK-21 cells using a 96-well tissue culture plate format. Five molecules belonging to different chemical classes were identified and showed >90% inhibitory activity against RABV. Among them, the hit molecule, HHT, was further characterized against CVS-11 and clinical isolates of RABV in BHK-21 and Neuro-2a cell lines.

## 3. Materials and Methods

### 3.1. Cell Lines

BHK-21 and Neuro-2a (mouse neuroblastoma) cells were cultured in Eagle’s minimum essential medium (EMEM) supplemented with 10% fetal bovine serum (FBS), an antibiotic antimycotic solution containing penicillin (100 units/mL), streptomycin (100 µg/mL) and Amphotericin B (0.25 µg/mL) at 37 °C in a humidified CO_2_ (5%) incubator. All cell culture reagents were obtained from Sigma-Aldrich, St. Louis, Missouri, USA.

### 3.2. RABV (CVS-11) and Clinical Isolates of RABV

The laboratory adapted RABV strain (CVS-11) was propagated in the BHK-21 cells. In brief, the BHK-21 cells were infected with a multiplicity of infection (MOI) of 0.01 in EMEM containing 2% FBS and incubated at 37 °C in a humidified CO_2_ (5%) incubator for 48 h. After 48 h incubation, the supernatant was collected, and the cell debris was removed by centrifugation. The virus stocks were titrated using the gold standard direct fluorescence antibody (DFA) assay as described previously using FITC-conjugated anti-rabies monoclonal antibody [23,24,25] targeting RABV N protein (Fujirebio Diagnostic Inc., Ghent, Belgium) and stored in aliquots at −80 °C.

Clinical isolates of RABV were obtained by isolating the virus from the brain samples of rabies-infected animals. Initially, these samples were confirmed for rabies infection via lateral flow assay (LFA) and DFA (reports of KVAFSU-CVA Rabies Diagnostics Lab, Dt. 10.08.2022, 23.12.2022, 29.08.2023 and 09.10.2023). Brain samples from jackal (VMC 2124 and VMC 2611), lion (VMC 2219) and dog (VMC 2577) were preserved in the repository at the KVAFSU-CVA Rabies Diagnostic Laboratory, WOAH Reference Laboratory, Veterinary College, KVAFSU, Bengaluru, India. In brief, a small piece of the brain tissue was cut and homogenized using a cell culture medium (DMEM). The homogenate was centrifuged at 300× *g* for 10 min to remove debris. The supernatant was collected and used as an inoculum for the propagation and adaptation of the rabies virus in the BHK-21 cell line. After serial passages, the adaptation of the virus to the BHK 21 cell line was confirmed by LFA, DFA, one-step PCR and a rapid fluorescent focus inhibition test (RFFIT). The titer of the BHK-21 adapted rabies viral isolates was determined and then preserved at −80 °C until further use.

### 3.3. FDA-Approved Drug Library

A unique collection of FDA-approved drug library, comprising 3035 diverse compounds, was obtained from SelleckChem (Cat # L1300-Z558271; Selleckchem.com). The stock solutions were prepared either in DMSO or water and received in a 96-well plate format. The plates were preserved at −20 °C until further use. The primary antiviral screening of compounds was done at a 10 µM concentration using the validated flow cytometry assay.

### 3.4. Optimization of a High-Throughput Flow Cytometry Assay

The optimum MOI, incubation time, and antibody dilution for the high-throughput assay were determined by titration in a 96-well tissue culture plate (Nest Biotechnology, Woodbridge, NJ, USA). For an optimum FITC antibody dilution, a preformed monolayer of BHK-21 cells was infected with RABV (CVS-11) in EMEM (2% FBS) at an MOI of 0.5 and incubated for 24 h at 37 °C in a CO_2_ incubator. After 24 h incubation, the cells were stained using FITC-conjugated anti-RABV antibodies at different dilutions in PBS, such as 1:50, 1:100, 1:150 and 1:200, and cells positive for RABV infection were determined through flow cytometry. The fluorescent intensity (% positive cells) of stained cell control and virus control was compared at each dilution to determine the optimum dilution at which the cell control shows negligible fluorescence compared to the virus control, which shows maximum fluorescence intensity. Further, the optimum MOI and incubation time were determined by infecting the preformed monolayer of BHK-21 cells with RABV at different MOIs in EMEM (2% FBS), such as 0.01, 0.02, 0.05, 0.1, 0.2 and 0.4, and incubating at 37 °C in a CO_2_ incubator for 24 h, 48 h and 72 h. Post-incubation, the plates were processed at individual time points and stained with an optimum FITC-conjugated antibody dilution to determine the positive cells by flow cytometry.

**Figure 1:**
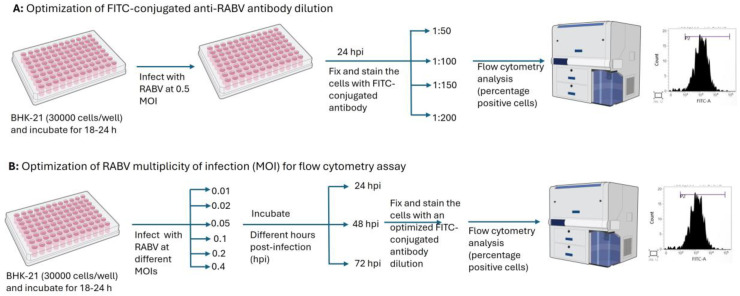
Schematic representation of (**A**) optimization of anti-RABV antibody dilution and (**B**) optimization of MOI for flow cytometry assay development. Created with NIH BIOART (NIAID Visual & Medical Arts. (10/8/2024). 96 Well Plate and Flow Cytometer. NIAID NIH BIOART Source. bioart.niaid.nih.gov/bioart/7 accessed on 24 June 2025, and bioart.niaid.nih.gov/bioart/160 accessed on 24 June 2025).

For flow cytometry analysis, the inoculum from the plates was removed after incubation; the cells were washed with PBS and then trypsinized for 5 to 10 min. This was followed by the addition of FBS at a final concentration of 10%. The cells were washed with PBS and fixed using 4% paraformaldehyde for 30 min. The fixed cells were washed twice with BD perm/wash buffer (BD Biosciences, San Diego, CA, USA) and stained with FITC-conjugated anti-RABV antibodies at 37 °C for 30 min. After incubation, the cells were washed twice with BD perm/wash buffer and suspended in PBS (100 µL/well). Later, the cells were analyzed in a flow cytometer (BD FACSLyric™ System) to detect the percentage of RABV-infected cells (positive cells). The percentage of positive cells from each well was determined by reading 15,000 cells per well. The cell control (without infection) was maintained as a negative control. The schematic representation of the assay development is given in Figure 1.

### 3.5. Development and Validation of a High-Throughput Antiviral Assay

**Figure 2:**
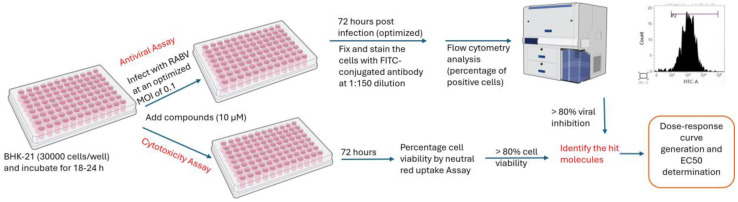
Schematic representation depicting the development of a flow cytometry-based high-throughput antiviral assay and cytotoxicity study. Created with NIH BIOART (NIAID Visual & Medical Arts. (10 August 2024). 96 Well Plate and Flow Cytometer. NIAID NIH BIOART Source. bioart.niaid.nih.gov/bioart/7 and bioart.niaid.nih.gov/bioart/160).

A flow cytometry-based high-throughput antiviral assay was developed in 96-well cell culture plates using FITC-conjugated anti-RABV monoclonal antibody targeting the N protein. The preformed BHK-21 monolayer in a 96-well culture plate was washed with PBS, infected with an optimized MOI (0.1) in EMEM (2% FBS) containing compounds at the desired final concentrations and then incubated for 1 h at 37 °C in a CO_2_ incubator for virus adsorption. After 1 h, the inoculum was removed, washed with PBS and compounds at the desired final concentrations were added in EMEM (2% FBS) to the respective wells in a final volume of 200 µL/well and incubated for an optimized time of 72 h. (hpi). After 72 h post-infection (hpi), the plates were processed for flow cytometry reading and analysis. Ribavirin and salinomycin were used as reference compounds to validate the assay [6,22,26]. The schematic diagram depicting the development of high-throughput assay and antiviral screening is given in Figure 2.

### 3.6. Antiviral Screening of FDA-Approved Drug Library

The primary screening of the library was done at the highest concentration of 10 µM using a flow cytometry-based assay. Before the antiviral assay, the cytotoxicity of the molecules was determined using a CyQUANT™ LDH Cytotoxicity Assay Kit (Invitrogen, Waltham, MA, USA) following the manufacturer’s instructions. The compounds non-cytotoxic at 10 µM were considered for primary antiviral screening. For the antiviral assay, the preformed BHK-21 monolayer was infected with RABV (CVS-11) at an MOI of 0.1 and incubated at 37 °C in a CO_2_ incubator for 1 h. After 1 h, the inoculum was removed, washed with PBS and compounds at the final concentration of 10 µM were added to the respective wells in a final volume of 200 µL/well. The plates were incubated at 37 °C in a CO_2_ incubator for 72 h (3 days). After incubation, the plates were processed to read the positive cells in a flow cytometer following the standard protocol (Section 3.5). The compounds with >50% viral inhibition were identified. The novel molecules exhibiting >90% viral inhibition were further characterized by establishing the dose-response curve (DRC) to determine the EC50 using flow cytometry and the conventional DFA method in different cell lines, such as BHK-21 and Neuro-2a.

The EC50 of the hit molecule (HHT) was determined by establishing the DRC with six concentrations (10 µM to 0.3 µM) prepared in two-fold serial dilutions. For flow cytometry, the cells were infected with RABV (CVS-11 or clinical isolates) at an MOI of 0.1 in the presence of the compound and incubated for 72 h. After 72 hpi, the cells were processed and analyzed in the flow cytometer to determine the EC50 of the compound. To determine the EC50 of HHT via the conventional DFA method, the cells were infected with RABV (CVS-11 or clinical isolates) at an MOI of 0.01 in the presence of HHT and incubated for 48 h. After 48 hpi, the supernatant was collected from each well, and the virus titer was estimated by DFA staining. The percentage reduction of RABV in the presence of HHT compared to the virus-only control was determined, and data were statistically analyzed to determine the EC50. Further, the cells were fixed and subjected to DFA staining using anti-RABV antibody in the presence of counterstain (Evans blue, MACKLIN, Shanghai, China). Microscopic pictures of the stained cells were captured (40X) to corroborate the data.

### 3.7. Time of Addition (ToA) and Cell-to-Cell Infection Assays

For ToA and cell–cell infection assays, the preformed monolayer of BHK-21 cells was prepared in 96-well plates/8-well slides. The following day, the cells were washed and infected with RABV (CVS-11) at an MOI of 0.1 or 0.01, and HHT at different concentrations (10 µM to 0.3 µM) was added at different time points. In the pretreatment/prophylactic mode, the cells were treated with HHT 24 h before infection (−24 h). In contrast, for the concomitant mode, HHT and virus were added simultaneously (0 h), and for post-infection, HHT was added at 24 hpi (+24 h). The infection medium was removed after virus adsorption, and cells were washed with PBS and supplemented with EMEM containing HHT at different concentrations (10–0.3 µM). However, the cells were supplemented with EMEM (without HHT) for the pretreatment mode. The cells were analyzed after 72 hpi through flow cytometry to determine the percentage of positive cells and antiviral activity of HHT. Simultaneously, the supernatant was collected after 72 hpi, and viral particles from the supernatant were measured by DFA and expressed as fluorescent foci units per mL (FFU/mL).

A cell–cell infection assay was performed to determine the spread of RABV in the infected cell monolayer, as described previously, with minor modifications [20,26]. The overlay medium was added to the infected cells with HHT (at a final concentrations from 10 µM to 0.3 µM) and incubated for 48 h. After 48 hpi, the cells were fixed and stained with anti-RABV antibody in the presence of counterstain (Evans blue). A cluster of fluorescent foci (green) was manually counted under the microscope from the whole well. Only fluorescent foci with more than two adjacent infected cells (indicating cell-to-cell spread) were considered. Percentage inhibition (cell–cell infection) in the treated well was determined compared to the virus-only control and statistically analyzed to determine the EC50. The overlay medium was prepared by mixing EMEM and carboxy methyl cellulose (2%) at equal proportions (1:1).

### 3.8. Antiviral Activity of HHT Against Clinical Isolates in BHK-21 and Neural Cell Lines

The antiviral activity of HHT against different clinical isolates in BHK-21 and Neuro-2a cells was determined through flow cytometry following the standardized protocol. For antiviral activity using the DFA method, a preformed monolayer of cell lines (BHK-21 and Neuro-2a cells) was infected with RABV at an MOI of 0.01 and incubated for 48 h at 37 °C in a CO_2_ incubator in the presence of HHT at 1.0 µM. After incubation, the cells were processed for flow cytometry analysis. For the DFA, cells were stained with FITC-conjugated anti-RABV antibody in the presence of counterstain (Evans blue) and observed under the microscope for FFU. Additionally, the supernatant was also collected from the wells for virus titration by the DFA method. The virus titer in the supernatant was measured via DFA staining to measure the inhibitory activity of HHT.

### 3.9. Statistical Analysis

The data generated in the present study were statistically analyzed using GraphPad Prism 10. The statistical significance to determine the optimum FITC antibody dilution was determined by two-way ANOVA followed by Tukey’s multiple comparisons test. The EC50 of the hit molecules was determined by nonlinear fit regression curve analysis. A *p*-value less than 0.05 was considered statistically significant. *p* values associated with each graph are indicated as follows: *, *p*-value < 0.05; **, *p*-value < 0.01; ***, *p*-value < 0.001; ****, *p*-value < 0.0001.

## 4. Results

### 4.1. Optimized Parameters of a High-Throughput Flow Cytometry Assay

**Figure 3:**
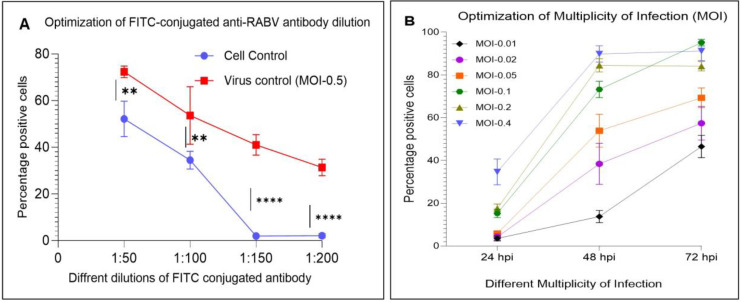
Optimization of a flow cytometry-based high-throughput antiviral assay. (**A**) Optimization of FITC-conjugated antibody dilution and (**B**) optimization of RABV (CVS-11) MOI. Optimum dilution of FITC-conjugated antibody and MOI required for high-throughput antiviral assay were determined in a 96-well plate using BHK-21 cells and RABV (CVS-11). The Z’ factor was calculated to determine the efficiency of the FITC-conjugated antibody dilution assay, and it was found to be 0.631 for a 1:150 dilution and 0.538 for a 1:200 dilution. **, *p*-value < 0.01; and ****, *p*-value < 0.0001.

The flow cytometry-based high-throughput assay was developed by optimizing the FITC-conjugated antibody dilution, multiplicity of infection, and incubation time. Two independent experiments (*n* = 2) with quadruplicates in each experiment were included to optimize the FITC-conjugated antibody dilution. The virus control with an MOI of 0.5 showed an average of 72.5% positive cells versus the cell control (without virus), which showed 52.17% at a 1:50 dilution of FITC antibody (Figure 3A). The FITC antibody dilution at 1:100 showed an average of 53.16% and 34.35% from virus and cell controls, respectively. However, the virus controls with the FITC-conjugated antibody dilution at 1:150 and 1:200 showed an average of 41.06% and 31.34% positive cells, respectively, versus the cell controls with 1.94% and 2.10%, respectively (Figure 3A). The FITC-conjugated antibody dilution at 1:200 showed a 10% reduction in the positive cells in virus controls compared to the 1:150 dilution. However, the cell controls showed a basal level positivity of about 2% in both 1:150 and 1:200 dilutions. The statistical analysis showed a significant difference between the virus and cell control at 1:150 and 1:200 dilutions with a *p*-value < 0.0001. However, the percentage of positive cells (RABV-infected) in the 1:200 dilution was found to be lower compared to the 1:150 dilution (Figure 3A). Furthermore, the Z’ factor at 1:150 is higher (0.631) compared to 1:200 (0.538), indicating the higher efficiency at 1:150 dilution. Hence, the FITC antibody dilution of 1:150 was considered the optimum for the detection of virus-infected (positive) cells in the assay.

To optimize the MOI and incubation time, the BHK-21 cells were infected with RABV (CVS-11) at different MOIs and incubated at various times post-infection. Two independent experiments (*n* = 2) with 6 replicates in each experiment were conducted to optimize the MOI and incubation time. Lower MOIs, such as 0.01, 0.02 and 0.05, showed an average infection of 46%, 55% and 68%, respectively, at 72 hpi (Figure 3B). The higher MOIs of 0.2 and 0.4 showed similar infection levels (84% for MOI, 0.2 and 90% for MOI, 0.4) at 48 hpi and 72 hpi (Figure 3B). The MOI of 0.1 showed 15%, 72% and 95% infection at 24 hpi, 48 hpi and 72 hpi, respectively. The MOI of 0.1 showed a gradual increase in the infection from 24 hpi and a peak infection of 95% at 72 hpi compared to other MOIs (Figure 3B). Hence, the MOI of 0.1 and incubation time of 72 hpi (3 days) were considered optimum for the antiviral assay development. The representative histograms of the flow cytometry assay for the optimization of FITC-conjugated antibody dilution and for 0.1 MOI are given in Supplementary Figures S1 and S2.

### 4.2. FITC Antibody-Based High-Throughput Antiviral Assay

The FITC antibody-based high-throughput antiviral assay was validated using reference compounds such as ribavirin and salinomycin. The EC50 values of ribavirin and salinomycin were found to be 10.08 µM (±1.9) and 0.07 µM (±0.0015), respectively, (Figure S3A,B) and are in corroboration with the previous studies [22,26]. Furthermore, comparing the DRC and EC50 values determined by flow cytometry and the conventional DFA method showed comparable results (Figure S4). Hence, the results obtained in the present study confirm the validation and robustness of the high-throughput antiviral assay.

### 4.3. Antiviral Hits from FDA-Approved Drug Library

**Figure 4:**
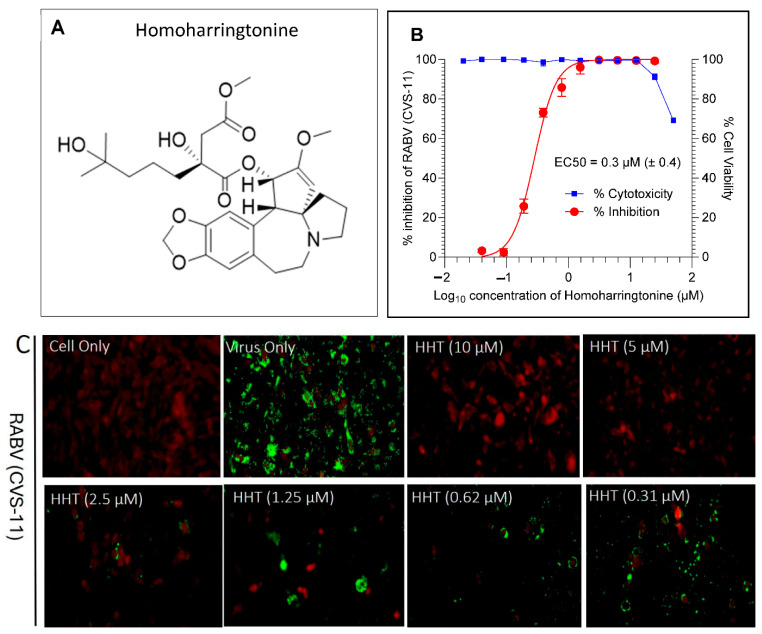
Dose-response curve (DRC) generation. (**A**) structure of HHT, (**B**) EC50 determination (*n* = 4) of HHT in concomitant assay (HHT was added along with infection) via flow cytometry and (**C**) antiviral activity of HHT by conventional DFA assay in BHK-21 cells. The cells were fixed and stained with FITC-conjugated anti-RABV antibody. The RABV appears green; the cells stained with Evans blue appear red (counterstain). Representative photomicrographs were taken after 48 h of incubation. The EC50 was determined through flow cytometry using the standard protocol. For the conventional DFA test, virus titer in the supernatant was determined by DFA staining to measure the EC50, and cells were fixed and stained to corroborate the data.

The primary antiviral screening identified 51 molecules with greater than 50% inhibition against RABV (Supplementary Table S1). The chemical diversity of these hit molecules is illustrated in Supplementary Figure S5. Five compounds, such as clofazimine, tiamulin, difloxacin, harringtonine and homoharringtonine, showed >90% inhibition at 10 µM. An anticancer drug, HHT (Figure 4A), was identified as a novel molecule with 92% RABV inhibition at 10 µM. The reported molecules, such as dasatinib, clofazimine and pyrimethamine, showed 72%, 94%, and 55% viral inhibition at 10 µM, respectively. Furthermore, the ribavirin, a part of the compound library, showed 58% activity at 10 µM. Among the five active compounds, HHT was prioritized based on the literature reports, chemical feasibility, and key properties such as blood–brain barrier permeability and established human use.

HHT and harringtonine share a similar core structure, with HHT differing by the presence of an additional methyl group in its ester side chain. Hence, HHT was further characterized for its antiviral activity against RABV. HHT showed an EC50 value of 0.3 µM (± 0.4) against RABV (CVS-11) in BHK-21 cells using flow cytometry assay (Figure 4B). Further, the conventional DFA assay in BHK-21 cells confirmed the activity of HHT with a similar EC50 (Figure 4C).

### 4.4. HHT Inhibits RABV at the Later Stages of Infection and Prevents Cell–Cell Spread

**Figure 5:**
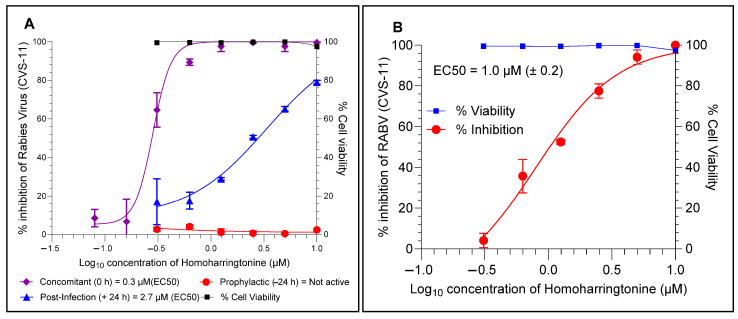
Time of addition assay (ToA) and cell–cell infection assay in BHK-21 cells. (**A**) HHT at given concentrations was added at different time points such as 24 h before infection (prophylactic, −24 h), simultaneously with infection (concomitant, 0 h) and 24 h after infection (post-infection, +24 h). The EC50 was determined via flow cytometry for each condition. (**B**) Cell–cell infection assay; the BHK-21 cells were infected with RABV (CVS-11) in the presence of HHT and then processed for a cluster of fluorescent foci to be counted under the microscope after DFA staining. The 50% reduction of RABV (EC50) was determined compared to the virus control.

HHT (C_29_H_39_NO_9_), an anticancer agent, is a cytotoxic alkaloid from the coniferous *Cephalotaxus harringtonia* tree. The ToA assay with HHT showed that HHT inhibits the RABV at later stages of infection in BHK-21 cells. In the pre-treatment mode, the HHT showed no inhibitory activity when it was retracted after infection. While the concomitant (0 h) and post-infection (+24 h) treatment modes showed EC50 values of 0.3 µM and 2.7 µM, respectively (Figure 5A), the EC50 value in the post-infection mode is comparatively higher than in the concomitant mode. The EC50 values obtained by estimating the viral load from the supernatants of concomitant (0 h) and post-infection (+24 h) samples via conventional DFA staining showed similar EC50 values and corroborated with the results obtained from the flow cytometry assay. Furthermore, the cell–cell spread assay demonstrated that RABV infection was prevented from cell–cell spreading with an EC50 value of 1.0 µM (Figure 5B).

### 4.5. HHT Inhibits Clinical Isolates of RABV in BHK-21 and Neuro-2a Cell Lines

**Figure 6:**
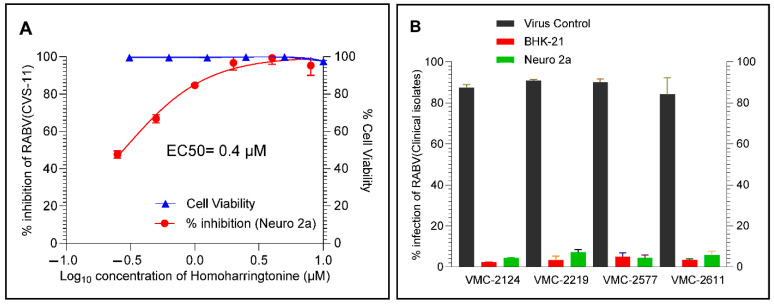
Antiviral activity of HHT against RABV (CVS-11) and clinical isolates. (**A**) Determination of EC50 of HHT against RABV (CVS-11) in Neuro-2a cells via flow cytometry and (**B**) determination of inhibitory activity of HHT against RABV (clinical isolates) in BHK-21 and Neuro-2a cells at 1.0 µM by flow cytometry.

**Figure 7:**
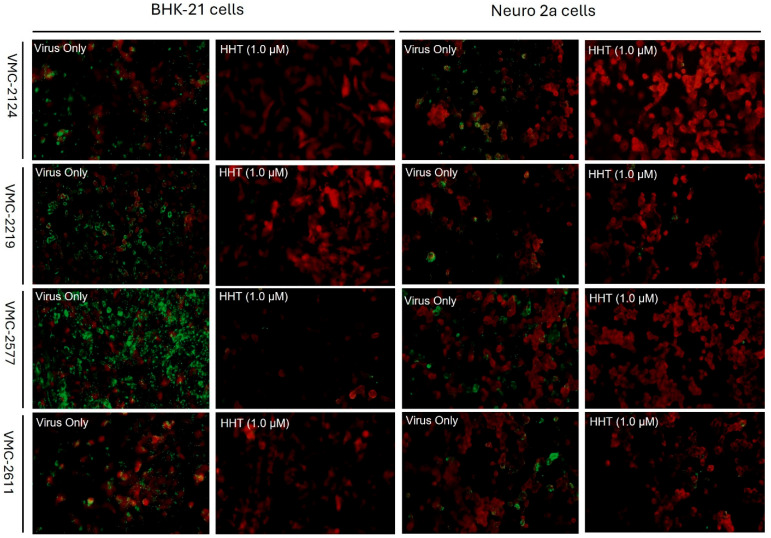
Antiviral activity of HHT against RABV (clinical isolates) by DFA staining. For the DFA assay, the cells (BHK-21 and Neuro-2a) were infected with RABV (clinical isolates) with or without HHT (1.0 µM) and incubated for 48 h. The cells were fixed and stained with FITC-conjugated anti-RABV antibody in the presence of counterstain Evans blue. Photomicrographs were taken; the RABV appears green, and the cells appear red (counterstain). In addition, the supernatant was collected from the wells for virus titration to corroborate the inhibitory activity of HHT.

The clinical isolates of RABV from the brain samples of infected animals were adapted to BHK-21 cells and confirmed by LFA, DFA, PCR and RFFIT. The flow cytometry assay showed an EC50 value of 0.4 µM against CVS-11 in Neuro-2a cells, comparable to its activity in BHK-21 cells (Figure 6A). Furthermore, the flow cytometry assay demonstrated inhibition of RABV clinical isolates (VMC 2124, VMC 2219, VMC 2577 and VMC 2611) by HHT in BHK-21 and Neuro-2a cells at 1.0 µM (Figure 6B). Additionally, the DFA assay also showed the inhibition of RABV clinical isolates (VMC 2124, VMC 2219, VMC 2577 and VMC 2611) in both BHK-21 and Neuro-2a cells at 1.0 µM and corroborates with the flow cytometry data (Figure 7).

## 5. Discussion

Rabies is an acute, fatal, progressive encephalomyelitis caused by RABV. RABV is a highly neurotropic virus that enters the nervous system from bite wounds through peripheral nerve synapses [27]. The virus reaches the central nervous system (CNS) by hijacking the retrograde axonal transport machinery and replicates exponentially [28]. Eventually, the prolonged replication of RABV in the brain leads to the onset of rabies clinical symptoms such as paralysis, anxiety and progressive, fatal encephalitis, leading to fatal disease progression [29]. The centrifugal spread of the virus in the peripheral nervous system (PNS) typically occurs just days before the onset of clinical signs in animals and humans [27]. The salivary glands act as portals of exit for the RABV into the saliva [30]. However, upon the appearance of clinical signs, rabies becomes 100% fatal, and patients die quickly due to the absence of therapeutics or intensive supportive care. Though rabies is a 100% vaccine-preventable disease, globally, human deaths due to rabies are significantly high. Hence, the development of antivirals against RABV is an urgent and unmet medical need.

Advanced techniques are being developed and used in clinical, immunological and infectious disease research, and the flow cytometry technique is one such technique. Many viruses have been detected using flow cytometry, including herpes simplex virus [31], cytomegalovirus [32] and rotavirus [33]. Furthermore, there are reports on using flow cytometry to detect intracellular RABV [34] and antigen or antibody detection [35]. However, the use of flow cytometry in rabies research and diagnosis is minimal despite its high sensitivity. The present FITC-conjugated antibody-based flow cytometry assay was validated and subsequently used in high-throughput antiviral screening against RABV. Few research groups have developed reporter RABV strains using reverse genetics and employ them in high-throughput antiviral screening [22,26]. However, the flow cytometry assay developed in the present study enables the screening of antivirals against laboratory-adapted strains and clinical isolates of RABV in various cell lines.

Repurposed antiviral drugs, such as ribavirin, favipiravir and interferon-alpha, have shown in vitro antiviral activity against RABV. However, they failed to show any beneficial activity in the in vivo studies [5,6,21]. The three active molecules (dasatinib, clofazimine and pyrimethamine) identified in the present study were reported to have antiviral activity against RABV in vitro but failed to show in vivo efficacy [5,36,37]. Ribavirin, a nucleoside analogue, is known to have in vitro activity against RABV (EC50 18.6 μM), but no activity is observed against clinical rabies, and it fails to protect the mouse model of infection [5,14,26]. A tyrosine kinase inhibitor, dasatinib, was picked up as a hit molecule in the present assay, exhibiting 84% inhibition at 10 µM. These data correlate with a previous study, which detected dasatinib as a hit molecule in a high-throughput assay with 93% inhibition at 10 µM [22]. This establishes the robustness of the present assay and its application in high-throughput screening of large chemical libraries against RABV.

HHT (C_29_H_39_NO_9_), a natural cytotoxic alkaloid derived from the *Cephalotaxus harringtonia* tree, is an anticancer agent that acts on ribosomes and inhibits protein synthesis in cancer cells [38]. In the present study, HHT demonstrated antiviral activity against a laboratory strain of RABV (CVS-11) with an EC50 value of 0.3 µM in BHK-21 cells. The mechanistic studies using the ToA assay showed late entry inhibition of RABV with an EC50 value similar to that of the concomitant assay. The absence of inhibitory activity in the prophylactic data indicates the lack of early entry inhibition. Furthermore, the cell-to-cell spread assay demonstrated the prevention of infection spread between cells after 48 hpi.

Additionally, the anti-RABV activity of HHT was established in mouse neural cell lines such as Neuro-2a. HHT demonstrated antiviral activity against RABV (CVS-11) in Neuro-2a cells, with an EC50 value similar to that observed in BHK-21 cell lines. Furthermore, HHT inhibited the clinical isolates of RABV in BHK-21 and Neuro-2a cells using both flow cytometry and DFA assays. This validates the use of flow cytometry in screening antivirals against different isolates of RABV in different cell lines. The antiviral data obtained from the conventional DFA assay corroborated the flow cytometry data. In summary, HHT inhibited the RABV (CVS-11 and clinical isolates) across all tested cell lines, including neural cell lines.

Earlier studies reported the broad-spectrum antiviral activity of HHT against RNA and DNA viruses, including mouse hepatitis coronavirus, porcine epidemic diarrhea virus (PEDV), vesicular stomatitis virus (VSV), Newcastle disease virus (NDV) and herpes simplex virus type 1 (HSV-1) [39,40]. Furthermore, a study by Gong et al. demonstrated the antiviral activity of HHT against foot-and-mouth disease virus [41]. Additionally, HHT is also reported to inhibit viral replication of a pseudorabies virus (PRV), an etiological agent of pseudorabies or Aujeszky’s disease [39]. A previous study revealed that HHT inhibits the early phases of FMDV replication (post-entry inhibition) [41]. A study by Dong et al. demonstrated the attenuation of the phosphorylation level of eukaryotic initiation factor (eIF4E) by HHT during PEDV, HSV-1 and PRV infection, which exhibited an inhibitory effect on viruses [39,42]. Viral infections lead to hijacking of the host cell machinery by the virus for replication and translation. Several studies showed the manipulation of eIF4E and its regulatory cellular proteins during viral infections. The viral-induced mechanisms in eIF4E are diverse and impact viral protein synthesis, the replication cycle, stimulation of viral reactivation and even proliferation of infected cells [42]. Translation and replication of murine coronavirus and herpesvirus HSV-1 require phosphorylation of eIF4E [43,44]. In contrast, during the influenza virus and VSV infection, dephosphorylation of eIF4E occurs [45,46]. However, the matrix protein of RABV favors viral mRNA translation and inhibits host translation by interacting with eIF3H [47]. The results obtained in the present study can be attributed to the post-entry inhibition of RABV by HHT.

HHT has been reported to exhibit broad-spectrum antiviral activity in vitro, and the present study investigates its antiviral activity against RABV, an etiological agent of rabies, a neglected tropical disease (NTD). However, an in vivo study against RABV could help develop HHT as a potential therapeutic against rabies.

In conclusion, we demonstrated the antiviral activity of HHT against CVS-11 and clinical isolates of RABV in different cell lines, including neural cells, in a time and dose-dependent manner. The availability of phase I/II clinical trials (safety and toxicity) data of HHT from cancer investigations supports repurposing HHT to expedite the development of a repurposed rabies therapeutic to meet the unmet medical needs of hospitalized patients.

## 6. Published Material

This article is a revised and expanded version of a paper entitled “Homoharringtonine Inhibits Rabies Virus In Vitro”, which was presented at the 38th International Conference on Antiviral Research (ICAR), Las Vegas, Nevada, from 17 March to 21 March 2025 [48].

## Data Availability

Data will be made available on request.

## Supplementary Materials

The following supporting information can be downloaded at: https://www.mdpi.com/article/10.3390/v17070945/s1, Figure S1: Optimization of FITC-conjugated anti-rabies antibody, Figure S2: Representative histograms at 0.1 MOI, Figure S3: Validation of flow cytometry assay through dose response curve (DRC) generation and determination of EC50, Figure S4: Antiviral activity of ribavirin against RABV (CVS-11) by DFA staining, Figure S5: Diversity of the active molecules identified from the SelleckChem FDA-approved library with >50% activity against rabies virus, Table S1: Active compounds identified from the SelleckChem HTS Library with >50% antiviral activity at 10 µM.
