# Supplementary material for: Homoharringtonine Inhibits CVS-11 and Clinical Isolates of Rabies Virus In Vitro: Identified via High-Throughput Screening of an FDA-Approved Drug Library
Source: Viruses. 2025 Jul 4;17(7):945. doi: 10.3390/v17070945 (PMC12299688; doi:10.3390/v17070945)
Supplement: Supplementary file 1 [file viruses-17-00945-s001.zip › Supplementary Figures.pdf]

Supplementary Figures:

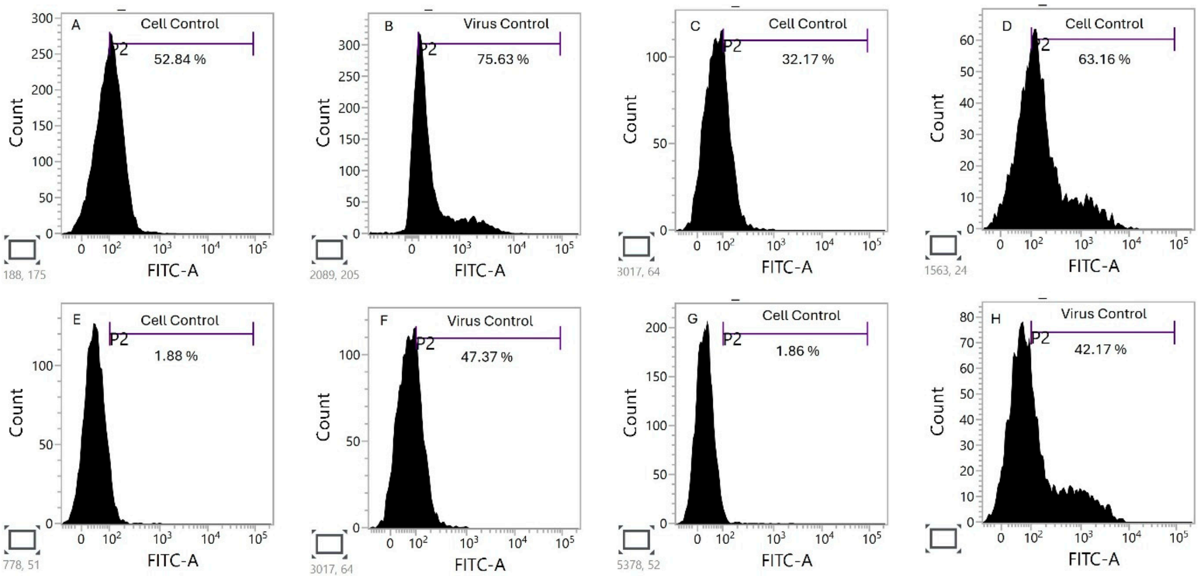

Figure S1: Optimization of FITC-conjugated anti-rabies antibody: Representative histograms obtained from BD FACSLyric™ System. For the optimum FITC antibody dilution, the preformed monolayer of BHK-21 cells was infected with RABV (CVS-11) at an MOI of 0.5 and incubated for 24 h at 37°C in a CO_2 incubator. After 24 h incubation, the cells were stained using FITC-labelled anti-RABV antibodies at different dilutions in PBS, such as 1:50, 1:100, 1:150, and 1:200, and cells positive for RABV infection (positive cells; P2) were determined by flow cytometry. **A** and **B**; 1:50 dilution, **C** and **D**; 1:100 dilution, **E** and **F**; 1:150 dilution, and **G** and **H**; 1:200 dilution.

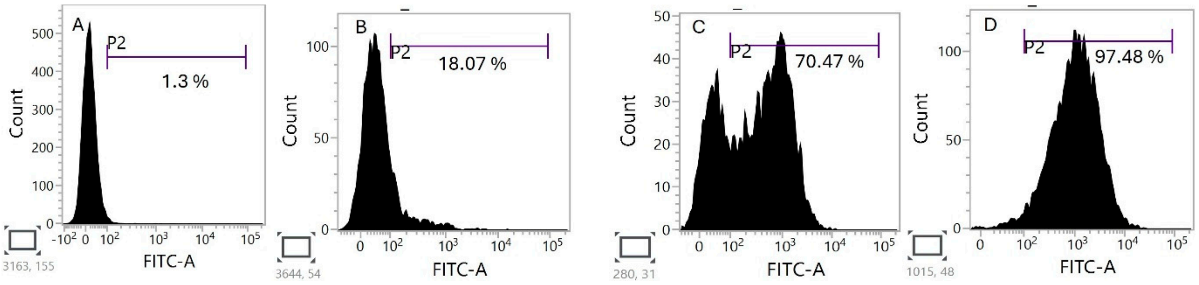

Figure S2: Representative histograms at 0.1 MOI: The preformed monolayer of BHK-21 cells was infected with RABV (CVS-11) with 0.1 MOI and incubated for 24 h, 48 h, and 72 h at 37°C in a CO_2 incubator. At respective post-incubation times, the cells were stained using FITC-

labelled anti-RABV antibodies at a 1:150 dilution, and cells positive for RABV infection (positive cells; P2) were determined by flow cytometry. **A,** Cell only, **B, C,** and **D**; virus controls at 24 h, 48 h, and 72 h post-infection, respectively.

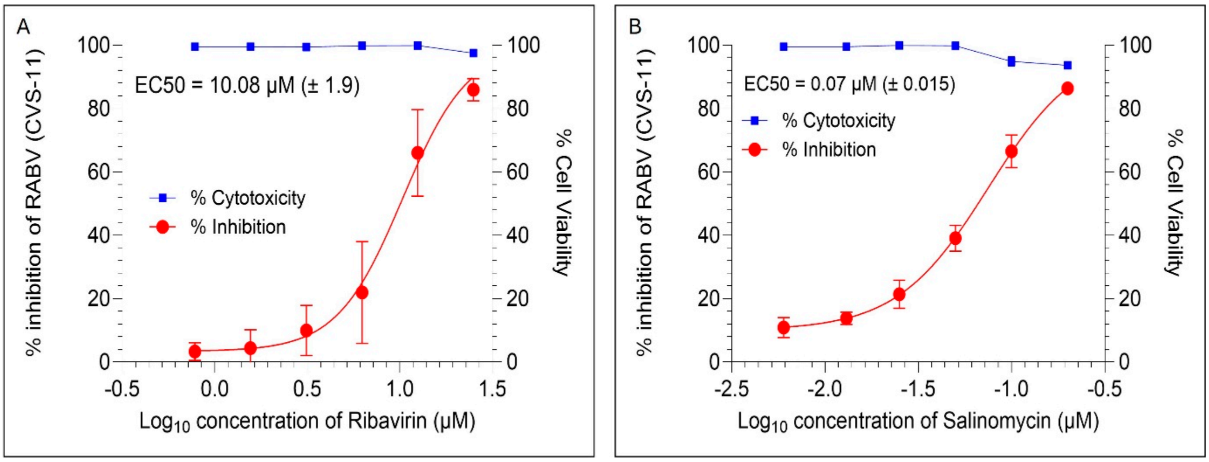

Figure S3: Validation of flow cytometry assay through dose response curve (DRC) generation and determination of EC50 of **A;** ribavirin and **B;** salinomycin against RABV (CVS-11).

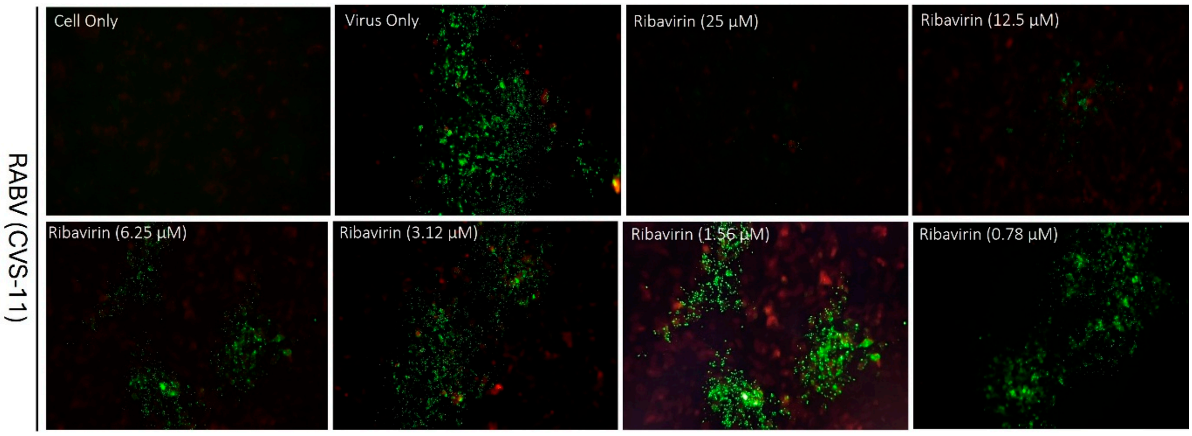

Figure S4: Antiviral activity of ribavirin against RABV (CVS-11) by DFA staining: BHK-21 cells were infected with RABV (CVS-11) with/without ribavirin and incubated for 48 hours. After incubation, the cells were fixed and stained with FITC-conjugated anti-RABV antibody, and representative photomicrographs were taken. Additionally, the supernatant was collected, and the virus titer was estimated to determine the EC50. The RABV stained with FITC-conjugated anti-RABV antibody appears green, and the cells stained with Evans blue appear red (counterstain).

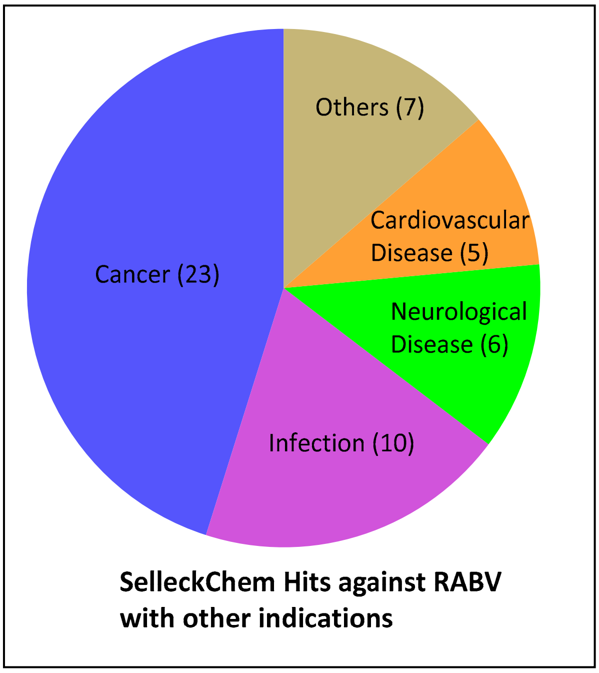

Figure S5: Diversity of the active molecules identified from the SelleckChem FDA-approved drug library with >50% activity against rabies virus
