# Supplementary material for: Homoharringtonine Inhibits CVS-11 and Clinical Isolates of Rabies Virus In Vitro: Identified via High-Throughput Screening of an FDA-Approved Drug Library
Source: Viruses. 2025 Jul 4;17(7):945. doi: 10.3390/v17070945 (PMC12299688; doi:10.3390/v17070945)
Supplement: Supplementary file 1 [file viruses-17-00945-s001.zip › Supplementary Table.pdf]

Supplementary Table S1

Sl. No.	Compound	Indication and pathway		Percentage inhibition of RABV (CVS-11)	Percentage cytotoxicity
1	Ridaforolimus (Deforolimus, MK-8669)	Cancer	PI3K/Akt/mTOR	61.52	0
2	Rapamycin (Sirolimus)	Immunology	PI3K/Akt/mTOR	62.13	0
3	Masitinib (AB1010)	Cancer	Protein Tyrosine Kinase	58.77	20
4	Gefitinib (ZD1839)	Cancer/Respiratory Disease	Protein Tyrosine Kinase	62.29	0
5	Docetaxel	Cancer/Respiratory Disease	Cytoskeletal Signaling	62.73	0
6	Finasteride	Endocrinology	Endocrinology & Hormones	61.08	0
7	Fluorouracil (5-Fluoracil, 5-FU)	Cancer	DNA Damage	67.81	0
8	Selumetinib (AZD6244)	Cancer	MAPK	58.05	21
9	Vismodegib (GDC-0449)	Cancer/Respiratory Disease	Stem Cells & Wnt	72.76	0
10	Anastrozole	Cancer	Endocrinology & Hormones	56.74	0
11	Vincristine sulfate	Cancer/Hematology/Respiratory Disease	Cytoskeletal Signaling	56.9	18
12	Nintedanib (BIBF 1120)	Cancer/Respiratory Disease	Protein Tyrosine Kinase	60.46	0
13	Temsirolimus (CCI-779, NSC 683864)	Cancer	PI3K/Akt/mTOR	56.49	0
14	Capecitabine	Cancer	DNA Damage	73.73	0
15	Aprepitant	Gastroenterology	Others	67.37	0
16	Raloxifene HCl	Cancer/Metabolic Disease	Endocrinology & Hormones	55.21	20
17	Fludarabine Phosphate	Cancer/Hematology	DNA Damage	65.23	0
18	Dasatinib	Cancer/Hematology	Angiogenesis	71.78	0
19	Naftopidil DiHCl	Others	Neuronal Signaling	90.81	0
20	Daclatasvir (BMS-790052)	Infection	Proteases	70.12	0
21	Fludarabine	Cancer/Hematology	JAK/STAT	85.4	0
22	Sulfameter	Infection	Metabolism	58.72	0
23	Ketoprofen	Neurological Disease/Inflammation	Neuronal Signaling	74.85	0
24	Emtricitabine	Infection	reverse transcriptase	69.92	0
25	Pyrimethamine	Infection/Parasitology/Respiratory Disease	Metabolism	55.31	0
26	Taxifolin (Dihydroquercetin)	Cardiovascular Disease	Angiogenesis	72.11	0
27	Troxerutin	Angiology/Cardiovascular Disease	Others	56.25	0
28	Ursolic Acid	Cardiovascular Disease	Others	59.45	0
29	Yohimbine HCl	Neurological Disease	Neuronal Signaling	53.62	0

30	5-hydroxytryptophan (5-HTP)	Psychotic disorders	Others	53.6	0
31	Aloin	Gastroenterology	Proteases	59.22	0
32	Butylscopolamine Bromide	Neurological Disease	Others	66.66	0
33	Ribavirin	Infection	viral replication	57.33	0
34	Dehydroandrographolide Succinate Potasium Salt	Inflammation/Respiratory Disease	Immunology & Inflammation	51.92	0
35	Procaine HCl	Neurological Disease	Transmembrane Transporters	76.74	0
36	Clofazimine	Infection	Metabolism	94.54	0
37	Tiamulin	Infection	Others	92.02	0
38	Difloxacin hydrochloride	Infection	DNA replication	92.25	0
39	Pyridoxal phosphate	Nutritional support	Metabolism	73.77	0
40	DL-Menthol	Neurological Disease	Others	84.47	0
41	Amodiaquine	Infection/Parasite	Heme polymerase	55.90	20
42	Chlorhexidine diacetate	Infection	Cell Membrane	87.9	10
43	Monomethyl auristatin E (MMAE)	Cancer	Cytoskeletal Signaling	72.55	22
44	Harringtonine	Cancer	Others	95.83	0
45	Anlotinib (AL3818) dihydrochloride	Cancer/Gastroenterology/Respiratory Disease	Protein Tyrosine Kinase	75.86	10
46	Penbutolol Sulfate	Cardiovascular Disease	N/A	72.48	0
47	Bepridil hydrochloride	Cardiovascular Disease	Transmembrane Transporters	76.13	0
48	Vindesine sulfate	Cancer	Cytoskeletal Signaling	87.28	0
49	Homoharringtonine	Cancer	IL-6/JAK1/STAT3 signal pathway	91.72	0
50	Perhexiline maleate	Cardiovascular Disease	Metabolism	78.46	10
51	Bismuth Subcitrate Potassium	Infection	others	68.23	0

Supplementary Table S1: Active compounds identified from the SelleckChem HTS Library with >50% antiviral activity at 10 µM. Homoharringtonine was considered for further characterization.
